# H1N1 exposure during the convalescent stage of SARS-CoV-2 infection results in enhanced lung pathologic damage in hACE2 transgenic mice

**DOI:** 10.1080/22221751.2021.1938241

**Published:** 2021-06-13

**Authors:** Heng Li, Xin Zhao, Yurong Zhao, Jing Li, Huiwen Zheng, Mengyi Xue, Lei Guo, Jian Zhou, Jinling Yang, Yuanyuan Zuo, Yanli Chen, Zening Yang, Qiqi Fan, Li Qin, Haijing Shi, Longding Liu

**Affiliations:** Institute of Medical Biology, Chinese Academy of Medical Sciences and Peking Union Medical College, Kunming, People’s Republic of China

**Keywords:** SARS-CoV-2, H1N1, alternate infection, convalescent stage, hACE2 transgenic mice

## Abstract

The risk of secondary infection with SARS-CoV-2 and influenza A virus is becoming a practical problem that must be addressed as the flu season merges with the COVID-19 pandemic. As SARS-CoV-2 and influenza A virus have been found in patients, understanding the in vivo characteristics of the secondary infection between these two viruses is a high priority. Here, hACE2 transgenic mice were challenged with the H1N1 virus at a nonlethal dose during the convalescent stage on 7 and 14 days post SARS-CoV-2 infection, and importantly, subsequent H1N1 infection showed enhanced viral shedding and virus tissue distribution. Histopathological observation revealed an extensive pathological change in the lungs related to H1N1 infection in mice recovered from SARS-CoV-2 infection, with severe inflammation infiltration and bronchiole disruption. Moreover, upon H1N1 exposure on 7 and 14 dpi of SARS-CoV-2 infection, the lymphocyte population activated at a lower level with T cell suppressed in both PBMC and lung. These findings will be valuable for evaluating antiviral therapeutics and vaccines as well as guiding public health work.

## Introduction

Coronavirus disease 2019 (COVID-19) has become the most massive pandemic globally in the last one hundred years, consequently resulting in at least 57, 882,183 confirmed cases and 1,377,395 deaths worldwide as of 22 November 2020 [1]. In addition to facing the second and third waves of the SARS-CoV-2 epidemic this winter, we have to respond to the risk of merging the COVID-19 pandemic and the annual flu season, especially in the Northern Hemisphere.

In patients infected with SARS-CoV-2, more than 80% of patients have mild symptoms and good prognosis [2], and the fatality rate currently reported is approximately 2.9% [3]. Although COVID-19 has not caused high fatality rates, as a severe acute respiratory syndrome (SARS) (9–11%, 8098 cases and 774 deaths) [4] and the Middle East respiratory syndrome (MERS) did (34%, 2494 cases and 858 deaths) [5], the transmission rate of SARS-CoV-2 is obviously higher. Despite concerns focused on SARS-CoV-2, influenza continues to pose a tremendous threat to global public health, particularly during the winter months. Influenza activity was generally similar to that in previous seasons in most countries, and the influenza season can begin as early as October and peaks in January or February [6]. In addition to seasonal influenza causing approximately 1,000,000,000 and 650,000 deaths annually worldwide [7], we are threatened by sporadic infections by emerging avian influenza viruses, including highly pathogenic avian H5N1 [8] and H7N9 [9] viruses. Frustratingly, some patients have tested positive for both influenza virus and SARS-CoV-2 around the world and are infected with both viruses, SARS-CoV-2 and influenza A virus (IAV), in China [10], the USA [11], Italy [12], Iran [13], Singapore [14] and other countries.

Coinfection has been reported in patients with SARS [15,16] and MERS [17–19], and co-pathogens include bacteria, fungi and viruses, which lead to complications in the identification and treatment of respiratory syndrome and aggravate the state of illness. Microbial coinfection could increase the risk of disease severity in humans, and co-infection with H1N1 and SARS-CoV-2 has been reported in hamsters, and longer SARS-CoV-2 shedding and enhanced lung damage compared to that of SARS-CoV-2 mono-infection were found [20]. To date, there have been no reports about injury and immune responses in hosts infected with SARS-CoV-2 before infection with IAV, it is urgent to discuss the secondary infection between SARS-CoV-2 and IAVs in the flu season during the COVID-19 pandemic. Here, we report a hACE2 transgenic mouse model that was infected with SARS-CoV-2 at a nonlethal dose, and then, in the convalescent stage, the mice were challenged with H1N1 at a nonlethal dose. Enhanced infection was led by H1N1 exposure during the convalescent stage of SARS-CoV-2 infection in the hACE2 transgenic mice, with more serious injuries, higher virus loads, higher cytokines levels and lower lymphocytes levels were observed. These results and the mouse model with SARS-CoV-2 and H1N1 infection will be valuable for evaluating antiviral therapeutics and vaccines as well as guiding public health work.

## Materials and methods

### Viruses and cells

The viral strain SARS-CoV-2-KMS1/2020 (GenBank accession number: MT226610.1) was isolated from sputum collected from a COVID-19 patient by the Chinese Academy of Medical Sciences (IMBCAMS) and propagated and titrated on Vero cells in DMEM (Sigma, USA). The stock virus titre was 10^6^ cell culture infectious doses (CCID_50_)/ml, and frozen at −80°C prepared for all following experiments.

The H1N1 was mouse-adapted influenza virus A/PuertoRico/8/34 (H1N1).

### Animals and biosafety

All animal experiments were conducted under prior approval from the Animal Ethics Committee of the Institute of Medical Biology, IMBCAMS, according to the National Guidelines on Animal Work in China. Female hACE2 transgenic mice (ICR) were purchased from Wei Shang Li Tuo Technology Co., Ltd in Beijing, and they were generated by microinjection of the mice ACE2 promoter driving the human ACE2 coding sequence into the pronuclei of fertilized ova from ICR mice, and then human ACE2 integrated was identified by PCR as previously described [21], the hACE2 mainly expressed in lung, heart, kidney, and intestine of transgenic mice. The hACE2 ICR transgenic mice could be infected by SARS-CoV and SARS-CoV-2, and in Linlin Bao’s results [22], weight loss, virus replication and interstitial pneumonia in the lung were observed in hACE2 mice infected with 10^5^ CCID_50_ SARS-CoV-2, similar to initial clinical reports of pneumonia caused by SARS-CoV-2. In our results, the hACE2 ICR transgenic mice could be infected by 5 × 10^3^ CCID_50_ SARS-CoV-2 with virus replication, virus shedding and interstitial pneumonia.

All work with infectious SARS-CoV-2 was performed with approval under Biosafety Level 3 (BSL3) and Animal Biosafety Level 3 (ABSL3) conditions by the Institutional Biosafety Committee of Institute of Medical Biology (IMB) in Kunming National High-level Biosafety Primate Research Center.

### Viral challenge

**Figure 1.**
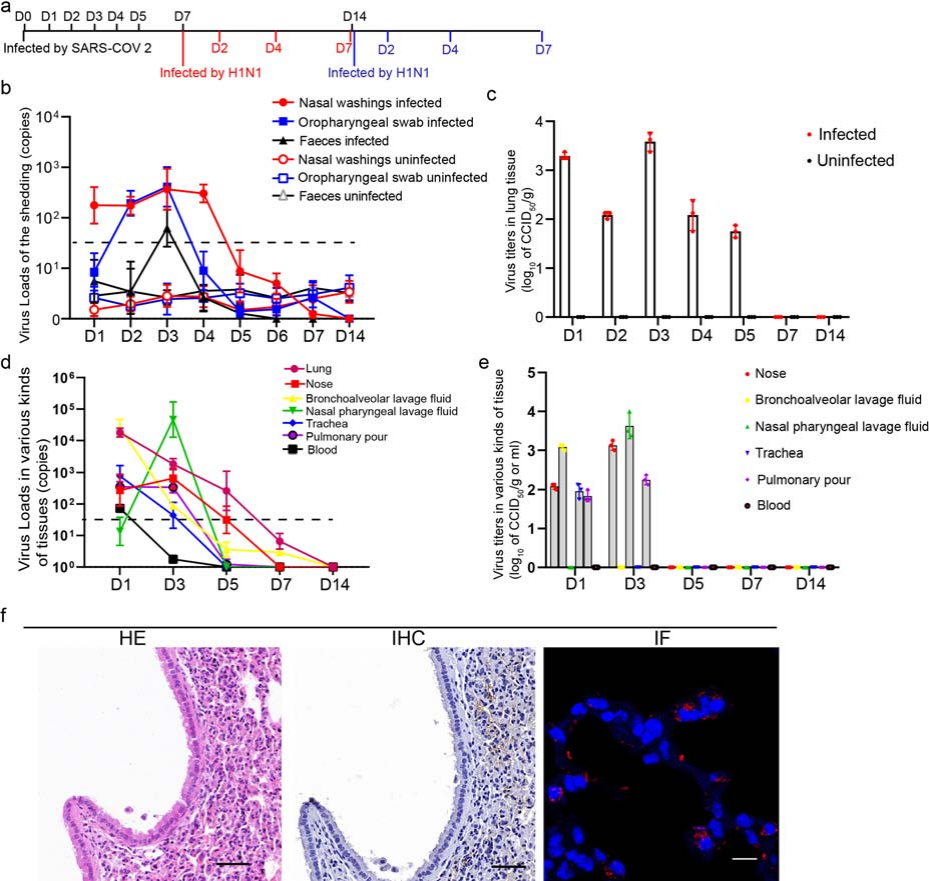
SARS-CoV-2 infection was established in hACE2 transgenic mice. (a) Time points of mouse sampled and challenged with SARS-CoV-2 and H1N1. (b) SARS-CoV-2 shedding in the nose, oropharynx and faeces were detected in the SARS-CoV-2-infected mice and uninfected mice. The samples were from 20 µl nasal washings, the whole oropharynx swabs and 100 mg faeces. (c) The SARS-CoV-2 virus titres in the lung were detected during SARS-CoV-2 infection in the SARS-CoV-2-infected mice and uninfected mice and peaked on 1 and 3 dpi after SARS-CoV-2 infection. The data were shown by log_10_ of CCID_50_ per 1 g lung tissue. (d) The SARS-CoV-2 virus loads in 100 mg or 100 µl tissues were detected in the trachea, nose, BALF, nasal pharyngeal lavage fluid, blood and pulmonary lymph node during SARS-CoV-2 infection. (e) The SARS-CoV-2 virus titres were detected in the trachea, nose, BALF, nasal pharyngeal lavage fluid, blood and pulmonary lymph node during SARS-CoV-2 infection. And the data were shown by log_10_ of CCID_50_ per 1 g or 1 ml tissues. (f) HE staining detection and IHC and IF by anti-SARS-CoV-2 nucleocapsid antibody in the lung of the mice on 3 dpi after infection with SARS-CoV-2, and the images in HE stains and IHC were captured from two continuous slides from the same lobe of the lung. The scales in HE and IHC were 50 µm, and the magnification factors were 20×; the scale was 10 µm, and the magnification factors were 630×. *n *= 3 in every point and the data were analysed using GraphPad Prism 8.

Six- to seven-week-old female hACE2 transgenic mice were used in this study. Nine mice were intranasally infected with 5 × 10^3^ CCID_50_ of SARS-CoV-2 (KMS1/2020 strain, GenBank accession number: MT226610.1) respectively, and the nasal washings, oropharyngeal swabs and faeces were collected every day. Nasal wash specimens were collected by washing with 20 µl of PBS; the oropharyngeal specimens were collected with cotton swabs, and about 100 mg of faeces specimens were collected. The mice were euthanized for tissue sampling on 1 day post-infection (dpi), 2, 3, 4, 5, 7 and 14 dpi. Nine mice were challenged with 100 CCID_50_ of H1N1 (A/Puerto Rico/8/34 strain) on 7 dpi after infection with SARS-CoV-2 and were euthanized and sampled on 2, 4 and 7 dpi after infected with H1N1. Nine mice were challenged with 100 CCID_50_ of H1N1 on 14 dpi after infection with SARS-CoV-2 and were euthanized and sampled on 2, 4 and 7 dpi after H1N1 infection (Figure 1(a))

### Virus load detection

Total RNA was extracted from oropharyngeal swabs, nasal washings, faeces, whole blood and homogenized tissues using TRIzol reagent (Tiangen, China). Viral genome RNA was detected quantitively by real-time PCR as copy numbers in 20 µl nasal washings, the whole swabs or 100 mg tissues. The primers and probes used were ORF1ab-F: 5’-CCCTGTGGGTTTTACACTTA-3’; ORF1ab-R: 5’-ACGATTGTGCATCAGCTG-3’; and ORF1ab-P: 5’-CCGTCTGCGGTATGTGGAAAGGTTATGG-3’. For quantitation of viral RNA, a standard curve was generated using 10-fold dilutions of RNA standard, the standard curve was *y *=* *−0.3402*x *+* *12.565. qRT-PCR was performed using a TaqMan Gene Expression Kit (Takara, China).

### Virus titre detection

The virus titres were determined by a micro-dose cytopathogenic efficiency (CPE) assay. Mixtures of ten-fold serially diluted tissue samples (from 10^0^ to 10^−7^) were incubated with 10^4^ Vero cells in 96-well culture plates. After 5 days of culture in a 5% CO2 incubator at 37°C, cells were checked for the presence of a CPE under a microscope. The virus titres were calculated by Karber’s methods. The equation was log_10_ of CCID_50_/0.1 ml = L + *d*(*S*−0.5), L was the logarithm of the lowest dilution multiple, *d* was coefficient of dilution (class interval), *S* was the sum of the cytopathic ratio.

### Histopathology

The specimens were fixed in 10% formalin for more than one week, and then the samples were fixed in 10% formalin for 2 h, 1 h in 70% ethanol, 1 h in 80% ethanol, 1 h in 90% ethanol, 1 h in 95% ethanol for 3 times, 1 h in xylene, 30 min in xylene, 30 min in paraffin, 1 h in paraffin for 2 times. After slicing, the sections of paraffin-embedded tissue were deparaffinized in xylene, rehydrated in a graded series of ethanol and rinsed with double-distilled water, and then, haematoxylin for 15 min, water for 1 min, 1% HCl in ethanol for 5 s, water for 1 min, ammonium hydroxide for 10 s, water for 1 min, 0.5% eosin for 30 s, 75% ethanol for 10 s, 95% ethanol for 10 s for 2 times, ethanol for 10 s for 2 times, xylene for 10 s for 2 times.

### Immunohistochemistry (IHC)

The sections of paraffin-embedded tissue were deparaffinized in xylene, rehydrated in a graded series of ethanol and rinsed with double-distilled water. The sections were incubated with rabbit anti-N antigen of SARS-CoV-2 for 1 h after heat-induced epitope retrieval. Antibody labelling was visualized by the development of DAB. Digital images were captured and evaluated by a histological section scanner (Aperio Digital Pathology, Leica).

### Immunofluorescence (IF)

Paraffin-embedded tissue sections were dewaxed, antigen repair was performed, the tissue slides were permeabilized with 0.1% Triton X-100 for 15 min and then, the tissue sections were blocked for 1 h in 5% BSA at room temperature (RT). And labelled with an anti-SARS-CoV-2 N protein antibody (Sino Biological, China) at 1:500 dilutions overnight at 4°C. Finally, SARS-CoV-2 N protein antigens were visualized by Alexa Fluor 647-conjugated goat anti-rabbit IgG at a 1:500 dilutions for one hour. The images were captured by a Leica TCS SP8 laser confocal microscope.

### Inflammatory cytokine and chemokine quantifications by RT-real-time PCR

The abundance of inflammatory cytokines and chemokines in the lungs were detected by RT-real-time PCR. 21 kinds of inflammatory cytokines and chemokines were performed, including IL-12, IL-17, IL-22, IL-33, MMP9, MIP1-α, PANTES, IFN-β, IFN-*γ*, IL-2, IL-4, IL-10, IL-6, MCP-1, CXCL-2, CXCL-13, IL-1 β, CXCL-1, CXCL-5, TNF-α and GM-CSF. The same lobe of the lung was harvested for total RNA extraction, and the total RNA were reverse-transcripted into cDNA for detection by real-time PCR, and the primer sequences were presented in sTable 2.

### Immunophenotyping

The lymphocytes abundance detection standards were by CD3 (PerCP/Cy5.5-conjugate), CD4 (PE-conjugate), CD19 (APC-conjugate) staining,Th1 cells: CD4+ IFN-γ+ (FITC-conjugated), TREG: CD4+ FOXP-3+(BV421-conjugated), Th2 cells: CD4+ IL-4 +(BV421-conjugated), and Th17 cells: CD4+ IL-17+ (FITC-conjugated) staining, neutrophile granulocyte cells: Ly-6G/Ly-6C+ (FITC-conjugated), Macrophage cells:CD3- F4/80+ (PE-conjugate), NK cells: CD3- CD49b+ (PE/Cyanine 7-conjugate). All of these antibodies were purchased from BD Biosciences, USA. Red blood cells were lysed using BD Pharm Lyse. The cells were permeabilized using BD permeabilization/fixation reagent. The cells were resuspended in 0.2 ml of 2% paraformaldehyde until they were run on a Beckman flow cytometer. For MHC tetramer assays, SARS-CoV-2 SPIKE MHC tetramer (KVGGNYNYL) (PE-conjugated) and influenza haemagglutinin MHC tetramer (IYSTVASSL) (APC-conjugated) were purchased from Creative Biosciences. Flow cytometry data were analysed using CytoFLEX (BECKMAN). The lymphocytes population were gated by FSC-A and FSC-H for removing the adherent cells.

### Statistical analysis

The data were analysed and plotted using GraphPad Prism 8, and *P-*values were calculated by one-way ANOVA using SPSS PASW statistical software version 18.0. *0.01 < *P* ≤ 0.05, **0.001 < *P* ≤ 0.01, and ***P ≤ 0.001.

## Results

### SARS-CoV-2 infection was performed in hACE2 transgenic mice

The mice were first infected by SARS-CoV-2, and the infectious progress was validated daily according to detection of viral shedding, viral RNA and infectious virus in tissues; detection of virus in situ by IF and IHC; and evaluation of lung injury by haematoxylin and eosin (HE) staining (Figure 1). SARS-CoV-2 shedding in the nose, oropharynx and faeces was detected (Figure 1(b)), and the viral load peaked on 3 dpi. The viral loads in the nose were higher than those in the oropharynx, and a transient viral shedding in faeces was detected on 3 dpi. The SARS-CoV-2 titres in the lung were detected from 1 to 5 dpi, peaking at 10^3.58^ CCID_50_/g on 3 dpi (Figure 1(c)). The virus extracted from the lung tissue homogenates, bronchoalveolar lavage fluid (BALF) and nasal pharyngeal lavage fluid of the mice on 3 dpi were inoculated on Vero cells and verified by RT–PCR (sFigure 1(b)). In addition, virus distribution was detectable in the trachea, nose, BALF and pulmonary lymph node on 1 dpi, while in the nose, nasal pharyngeal lavage fluid and pulmonary lymph node on 3 dpi (Figure 1(d,e)). No virus titres could be detected in the tissues on 5, 7 and 14 dpi. IHC and IF showed that there was abundant SARS-CoV-2 in the alveolar cells (Figure 1(f)), and were observed in the lung from 2 to 5 dpi (sFigure 3). Pathologically, inflammation infiltration and intervals thickened of lung alveolar were observed on 3 dpi, and recovered from 7 dpi (Figure 1(f), sFigure 2).

### SARS-CoV-2 infection disturbed the balance of lymphocytes in PBMC and lung

**Figure 2.**
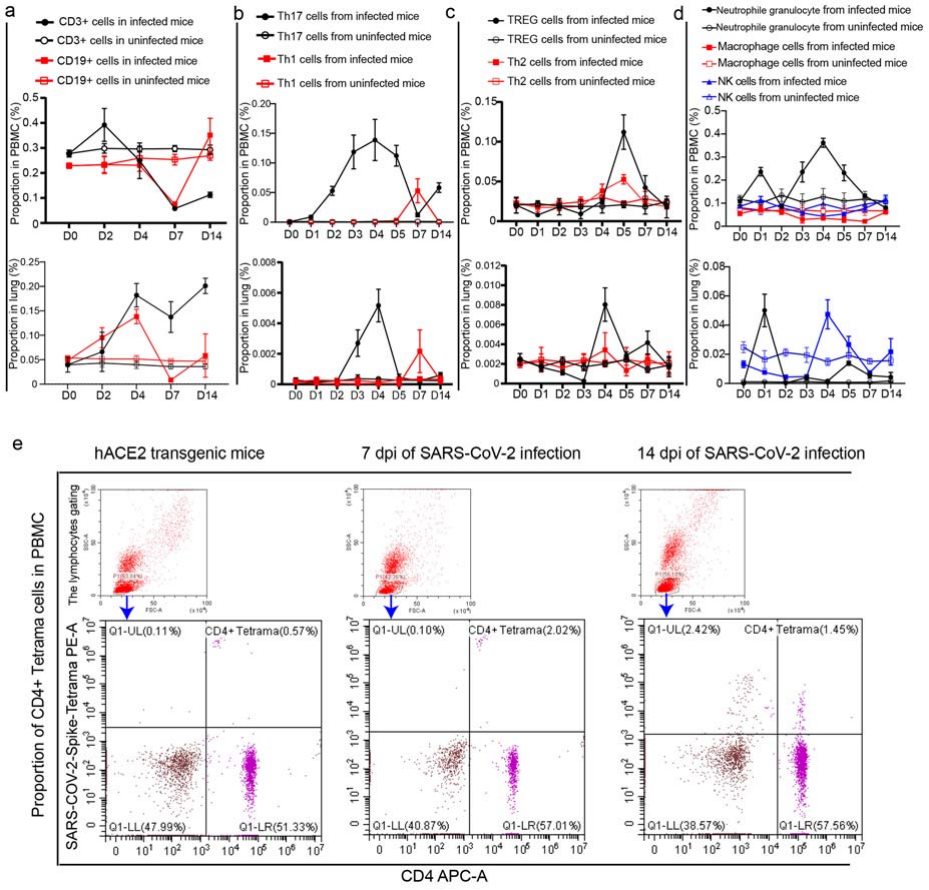
SARS-CoV-2 infection disturbed the balance of lymphocytes in PBMC and lung. (a) The proportions of CD3 and CD19 cells in PBMC and lung were analysed in the SARS-CoV-2-infected mice and uninfected mice. (b) The proportions of Th17 and Th1 cells in PBMC and lung were analysed in the SARS-CoV-2-infected mice and uninfected mice. (c) The proportions of Th2 and TREG cells in PBMC and lung were analysed in the SARS-CoV-2-infected mice and uninfected mice. (d) The proportions of innate lymphocytes including neutrophil granulocytes, macrophages and NK cells in PBMC and lung were analysed in the SARS-CoV-2-infected mice and uninfected mice. (e) CD4+ SARS-CoV-2-Spike-tetramer positive cells in PBMC were analysed during SARS-CoV-2 infection. *n *= 3 in every point and the data were analysed using GraphPad Prism 8.

During SARS-CoV-2 infection progress, CD3 cells, CD19 cells, natural killer (NK) cells and macrophages in PBMC of mice were suppressed; on the other hand, regulatory T (TREG) cells, Th1 cells, Th2 cells and Th17 cells could be stimulated in the infection process but maintained high levels for only a short time (Figure 2(a–d)). The proportion of CD3 cells showed increases in the early stage of SARS-CoV-2 infection but then suppressed until recovery (Figure 2(a)); CD19 cells were suppressed on 7 dpi until recovery (Figure 2(a)). For CD4 T cell subgroups, the proportion of Th17 and Th2 cells were observed increasing gradually from 1 and 3 dpi respectively, peaked on 4 and 5 dpi respectively. Notably, the abundance of Th1 and TREG cells were stable and increased only on 7 and 5 dpi respectively. All four CD4 T cell subgroups returned upon recovery. Neutrophil granulocytes showed increasing after infection, peaked on 1 and 4 dpi, and then returned until recovery (Figure 2(d)). Macrophages and NK cells were suppressed after infection and returned until recovery (Figure 2(d)). In the lung, the proportion of CD3 and CD19 cells showed increasing during in infection process, but the level of CD19 cells decreased on 7 dpi and returned to normal after recovery (Figure 2(a)). TREG cells, Th1 cells, Th17 cells response can be observed in the lung after infection with SARS-CoV-2 (Figure 2(b,c)), and Th17 and TREG cell levels peaked on 4 dpi, Th1 cells were detected only on 7 dpi, and no obvious change in the level of Th2 cells. TREG cells were also suppressed in the early infection stage and then proliferated on 4 dpi in the lung; neutrophil granulocytes were accumulated in the early infection stage, and then recovered; NK cells were stimulated, peaked on 4 dpi, and then recovered (Figure 2(d)). It should be noted that the proportions of CD3 cells and CD4 cells in PBMC of normal mice were significantly higher than those in PBMC of mice infected by SARS-CoV-2 for 7 and 14 days (sFigure 4). In addition, CD4+ SARS-CoV-2-Spike-tetramer cells in PBMC on 7 and 14 dpi were obviously higher than the levels before infection (Figure 2(e)).

### Enhanced infection, more serious lung injury was found in the H1N1-infected mice recovered from SARS-CoV-2 infection

**Figure 3.**
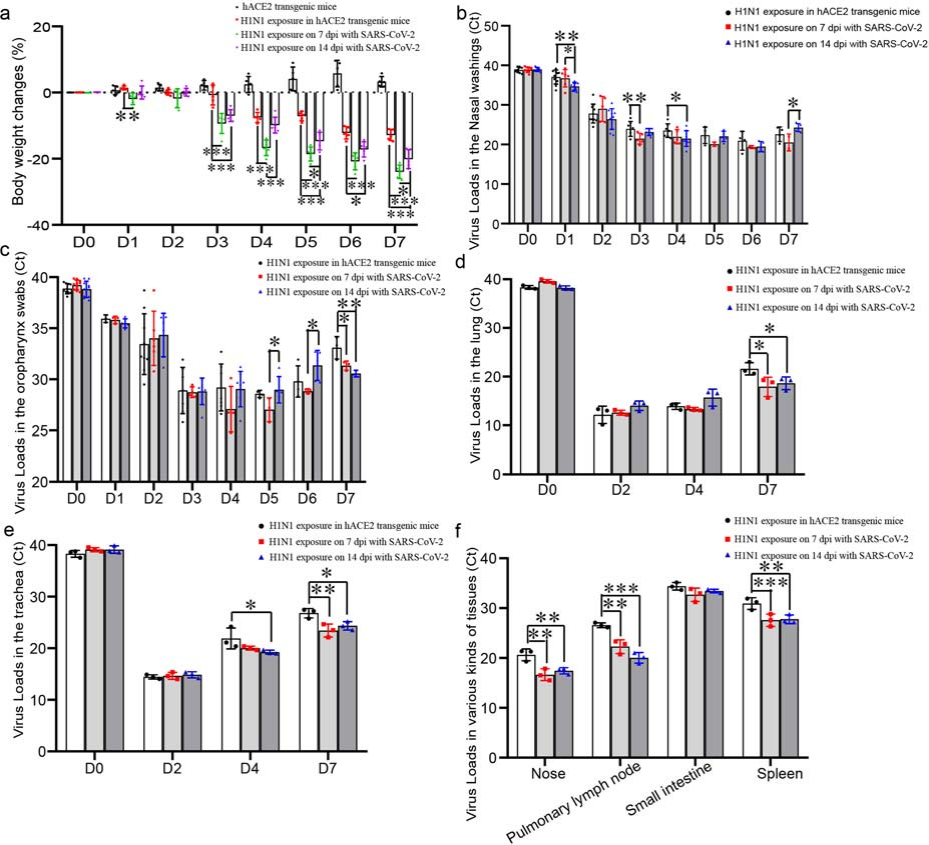
H1N1 infection was enhanced during rehabilitation from SARS-CoV-2 infection. (a) Weight changes in mice after challenged with H1N1, and the data were shown by change percentages. (b,c) The virus loads of H1N1 shedding in the nose (b) and oropharynx (c) were detected in the mice upon H1N1 exposure on 7 and 14 dpi with SARS-CoV-2 and in the mice without SARS-CoV-2 infection. The samples were from 20 µl nasal washings and whole oropharynx swabs, and the data were shown by cycle threshold (Ct) in the RT-real-time PCR. (d,e) The virus loads in the lung (d) and trachea (e) of the mice upon H1N1 exposure on 7 and 14 dpi with SARS-CoV-2 and in the mice without SARS-CoV-2 infection. The samples were from 100 mg tissues, and the data were shown by Ct in the RT-real-time PCR. (f) The virus loads on 4 dpi in the nose, pulmonary lymph node, small intestine and spleen of the mice upon H1N1 exposure on 7 and 14 dpi with SARS-CoV-2 and in the mice without SARS-CoV-2 infection. The samples were from 100 mg tissues, and the data were shown by Ct in the RT-real-time PCR. *n *=* *3 in every point and the data were analysed using GraphPad Prism 8, and the *P-*values were calculated by one-way ANOVA using SPSS PASW statistical software version 18.0. *0.01 < *P* ≤ 0.05, **0.001 < *P* ≤ 0.01, and ***P ≤ 0.001.

The mice infected with H1N1 maintained weight loss from 3 to 7 dpi, and the weight of the mice upon H1N1 exposure on 7 dpi of SARS-CoV-2 infection decreased most rapidly, whereas the weight of the mice upon H1N1 exposure without SARS-CoV-2 infection decreased most slowly (Figure 3(a)). In details, on 1 dpi after H1N1 infection, the weight loss was obviously more in the mice infected by SARS-CoV-2 for 7 days than ones in the mice without SARS-CoV-2 infection; on 3 and 6 dpi after H1N1 infection, the weight loss was obviously more in the mice infected by SARS-CoV-2 for 7 and 14 days than ones in the mice without SARS-CoV-2 infection; on 4 dpi after H1N1 infection, the weight loss was obviously more in the mice infected by SARS-CoV-2 for 7 days than ones in the mice without SARS-CoV-2 infection and the mice infected with SARS-CoV-2 for 14 days; on 5 and 7 dpi after H1N1 infection, the weight loss was most in the mice infected by SARS-CoV-2 for 7 days, and the weight loss was obviously more in the mice infected by SARS-CoV-2 for 14 days than ones in the mice without SARS-CoV-2 infection. H1N1 shedding in the nose and oropharynx and viral loads in various tissues of the mice, which infected with H1N1 in the SARS-CoV-2 infection convalescent stage, were higher than those in mice that were not infected with SARS-CoV-2 before infection with H1N1. H1N1 shedding in the nose on 1 dpi in the mice upon H1N1 exposure on 14 dpi of SARS-CoV-2 infection was significantly more than the shedding in the other two groups; furthermore, the H1N1 shedding on 3 dpi in the mice upon H1N1 exposure on 7 dpi of SARS-CoV-2 infection was significantly more than that in the mice without SARS-CoV-2 infection, and the shedding on 4 dpi in the mice upon H1N1 exposure on 14 dpi of SARS-CoV-2 infection was significantly more than that in the mice without SARS-CoV-2 infection (Figure 3(b)). H1N1 shedding in the oropharynx on 7 dpi in the mice upon H1N1 exposure after SARS-CoV-2 infection was significantly more than that in the mice without SARS-CoV-2 infection (Figure 3(c)). The H1N1 virus loads on 7 dpi in the lungs related to H1N1 infection in mice recovered from SARS-CoV-2 infection were significantly higher than those in the lungs of mice without SARS-CoV-2 infection (Figure 3(d)). The H1N1 virus loads on 7 dpi in the trachea related to H1N1 infection in mice recovered from SARS-CoV-2 infection were significantly higher than those in the mice without SARS-CoV-2 infection, and the virus loads on 4 dpi in the mice upon H1N1 exposure on 14 dpi with SARS-CoV-2 infection were significantly higher than those in the mice without SARS-CoV-2 infection (Figure 3(e)). The H1N1 virus loads on 4 dpi in the nose, pulmonary lymph node and spleen of the mice upon H1N1 exposure recovered from SARS-CoV-2 infection were significantly higher than those of the mice without SARS-CoV-2 infection (Figure 3(f)).

### Enhanced pathologic changes and more inflammatory cytokine and chemokine abundances in the lungs of H1N1-infected mice recovered from SARS-CoV-2 infection

**Figure 4.**
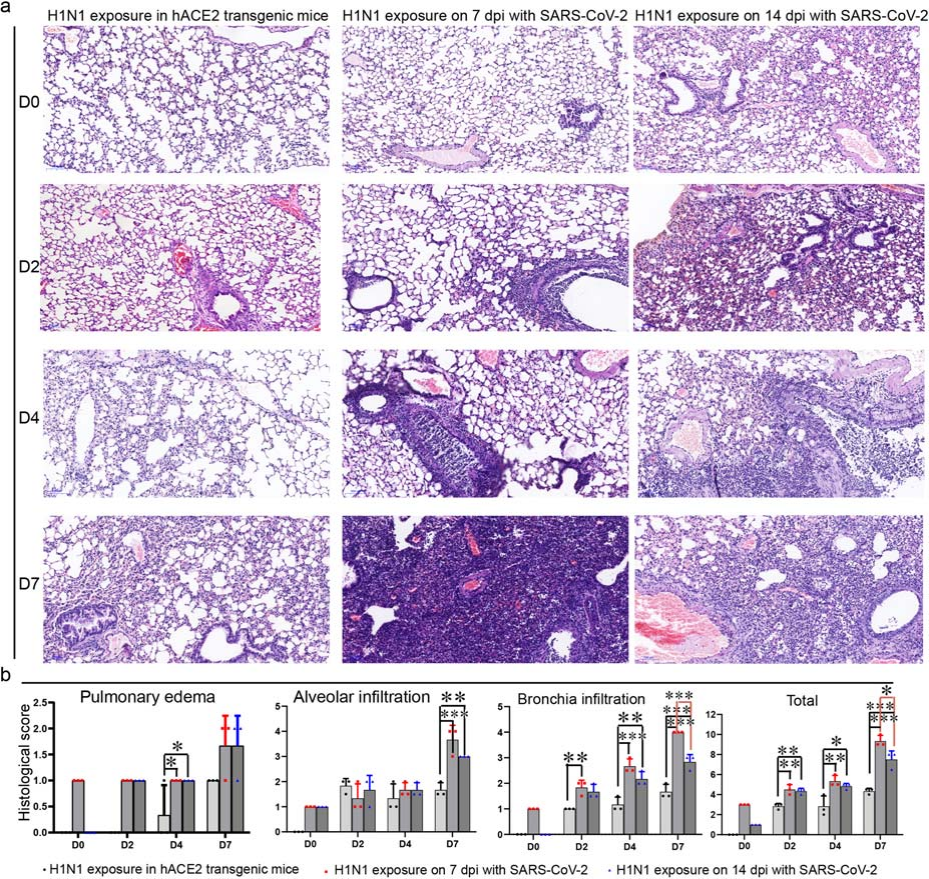
Lung injury by subsequent H1N1 infection was enhanced in the SARS-CoV-2 infection convalescent stage. (a) The lung injury by subsequent H1N1 infection were detected by HE staining, and in the early stage, inflammatory cells infiltrated around bronchioles or blood vessels, and then the cells in bronchi began to die and occlude the bronchi, in the last phase, large areas of pulmonary stroma were substantial lesions with inflammatory cell infiltration and alveolar cell shedding and death, and in the lungs of the mice without SARS-CoV-2 infection, the areas of substantial lesions were smaller than those in the mice infected with SARS-CoV-2 for 7 and 14 days. In addition, some alveolar septal cells thickened in the mice that had been infected with SARS-CoV-2. (b) Scoring of histopathological changes to quantify the lung injury, including Pulmonary oedema scores, Alveolar infiltration scores and Bronchia infiltration scores. The Histology score standards were in sTable 1. *n *= 3 in every point and the data were analysed using GraphPad Prism 8, and the *P-*values were calculated by one-way ANOVA using SPSS PASW statistical software version 18.0. *0.01 < *P* ≤ 0.05, **0.001 < *P* ≤ 0.01 and ***P ≤ 0.001.

Before challenging with H1N1, HE staining showed that the lungs in the SARS-CoV-2 infection convalescent stage on 7 and 14 dpi were basically normal except there were a few infiltrated inflammatory cells (Figure 4). After challenged with H1N1 on 7 and 14 dpi after SARS-CoV-2 infection for 2 days, inflammatory cells infiltrated around bronchioles or blood vessels, and a few cells in the bronchiole sloughed, and some alveolar septal cells thickened in the mice upon H1N1 exposure on 14 dpi of SARS-CoV-2 infection. Fewer inflammatory cells infiltrated around bronchioles or blood vessels in the lungs of mice without SARS-CoV-2 infection. After challenged with H1N1 for 4 days, the cells in all bronchi began to die and occlude the bronchi, and inflammatory cells infiltrated in bronchi and some pulmonary alveoli in the mice infected by SARS-CoV-2 before infection with H1N1; in the lungs of the mice without SARS-CoV-2 infection, the cells in some bronchi began to die and occlude the bronchi, and inflammatory cells infiltrated. After challenged with H1N1 for 7 days, large areas of pulmonary stroma were substantial lesions with inflammatory cell infiltration, alveolar cell shedding and death in the mice infected by SARS-CoV-2 before infection with H1N1, and the areas of substantial lesions in the mice infected with SARS-CoV-2 for 14 days were smaller than those in the mice infected with SARS-CoV-2 for 7 days. In the lungs of the mice without SARS-CoV-2 infection, the areas of substantial lesions were smaller than those in the mice upon H1N1 exposure in the SARS-CoV-2 infection convalescent stage (Figure 4, sFigure 5). Scoring of histopathological changes including Pulmonary oedema scores, Alveolar infiltration scores and Bronchia infiltration scores (sTable 2) also presented that most serious lung injury, especially for the bronchia infiltration was found in the mice infected by H1N1 after infected by SARS-CoV-2 for 7 days, and then more serious lung injury was found in the mice infected by H1N1 after infected by SARS-CoV-2 for 14 days than ones in the mice infected by H1N1 without SARS-CoV-2 infection (Figure 4(b)).

**Figure 5.**
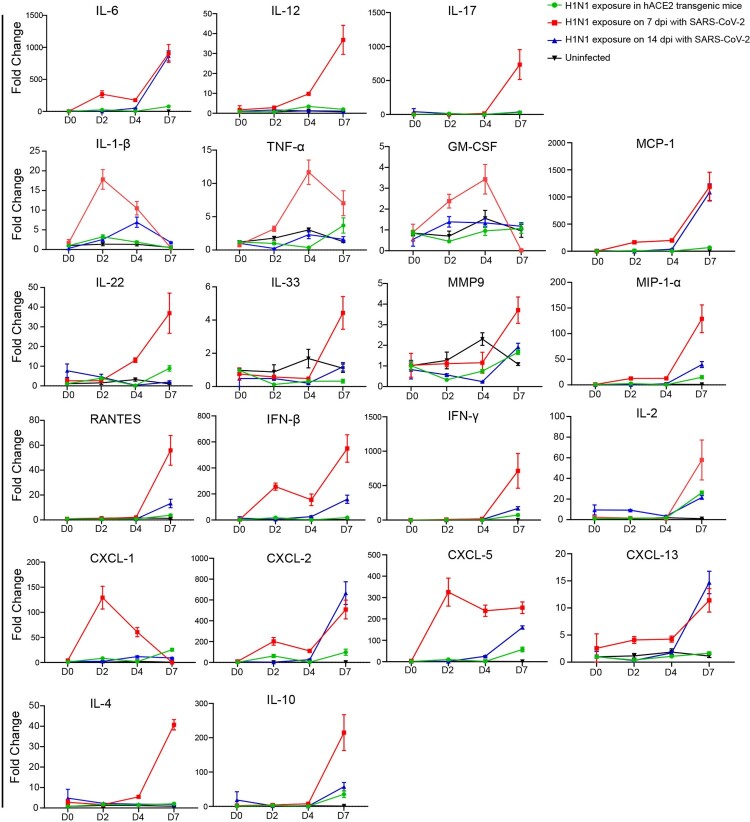
The abundance of inflammatory cytokines and chemokines by subsequent H1N1 infection in SARS-CoV-2 infection convalescent stage were enhanced The abundance of inflammatory cytokines and chemokines by subsequent H1N1 infection were detected by RT-real-time PCR in the four groups of mice including the mice upon H1N1 exposure on 7 and 14 dpi of SARS-CoV-2 infection and in the mice without SARS-CoV-2 infection was detected during H1N1 infection, the abundance of inflammatory cytokines and chemokines were also detected in the uninfected mice. The samples were from 100 mg tissues, and the data were shown by the fold changes of the abundance. Twenty-one kinds of inflammatory cytokines and chemokines were performed, including IL-12, IL-17, IL-22, IL-33, MMP9, MIP1-α, PANTES, IFN-β, IFN-*γ*, IL-2, IL-4, IL-10, IL-6, MCP-1, CXCL-2, CXCL-13, IL-1 β, CXCL-1, CXCL-5, TNF-α and GM-CSF, and the primer sequences were presented in sTable 2. *n *= 3 in every point and the data were analysed using GraphPad Prism 8.

In addition, 21 kinds of inflammatory cytokine and chemokine in the H1N1 infection were quantified in the lungs. The results demonstrated that the abundance of many pro-inflammatory factors such as IL-1 β, IL-6, TNF-α and GM-CSF; chemokines such as CXCL-1, CXCL-2, CXCL-5 and CXCL-13; antivirus cytokines such as IFN-β and IFN-*γ*; and anti-inflammatory factors such as IL-4 and IL-10 were higher on 7 dpi after H1N1 infection during the convalescent stage of SARS-CoV-2 infection. In details, the abundance of 11 kinds of them including IL-12, IL-17, IL-22, IL-33, MMP9, MIP1-α, PANTES, IFN-β, IFN-γ, IL-2, IL-4 and IL-10 were higher on 7 dpi after H1N1 infection in the mice with SARS-CoV-2 infection for 7 days than ones in the mice with SARS-CoV-2 infection for 14 days and the mice without SARS-CoV-2 infection; the abundance of 4 kinds of them including IL-6, MCP-1, CXCL-2 and CXCL-13 were higher on 7 dpi after H1N1 infection in the mice with SARS-CoV-2 infection for 7 days and 14 days than ones in the mice without SARS-CoV-2 infection; the abundance of three kinds of them including IL-1 β, CXCL-1 and CXCL-5 were higher on 2 dpi after H1N1 infection in the mice with SARS-CoV-2 infection for 7 days than ones in the mice with SARS-CoV-2 infection for 14 days and the mice without SARS-CoV-2 infection; the abundance of 2 kinds of them including TNF-α and GM-CSF were higher on 4 dpi after H1N1 infection in the mice with SARS-CoV-2 infection for 7 days than ones in the mice with SARS-CoV-2 infection for 14 days and the mice without SARS-CoV-2 infection (Figure 5).

### The levels of T cell responses were lower in PBMC and lung of H1N1-infected mice recovered from SARS-CoV-2 infection

**Figure 6.**
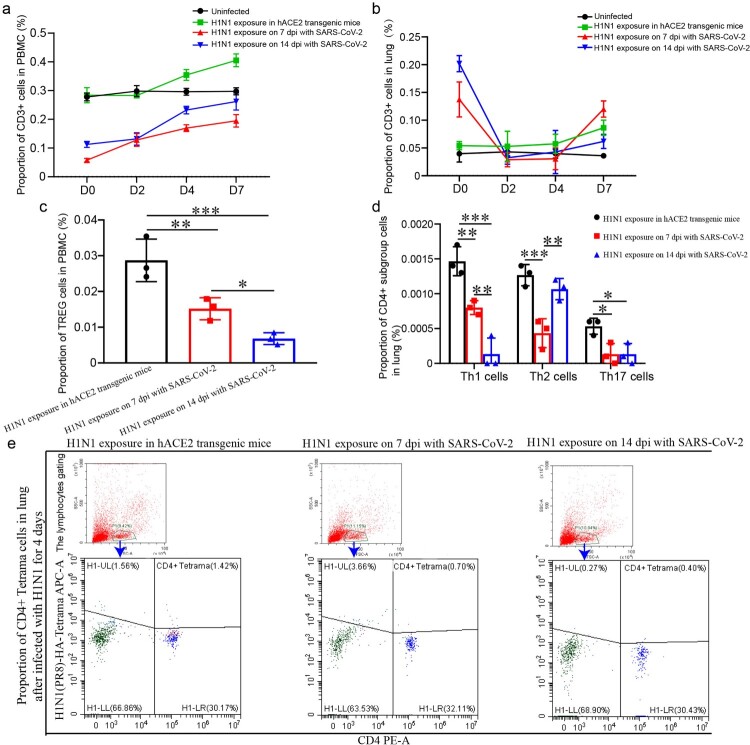
The levels of T cell responses were lower in PBMC and lung upon H1N1 infection during the convalescent stage of SARS-CoV-2 infection (a, b) CD3 cells in PBMC (a) and lung (b) in the mice upon H1N1 exposure on 7 and 14 dpi of SARS-CoV-2 infection and in the mice without SARS-CoV-2 infection were detected during H1N1 infection. The data were shown by percentages. (c) The level of TREG cells in PBMC on 4 dpi after H1N1 infection in the mice upon H1N1 exposure on 7 and 14 dpi of SARS-CoV-2 infection and in the mice without SARS-CoV-2 infection were showed. The data were shown by percentages. (d) The levels of Th17 cells, Th1 cells and Th2 cells in the lung on 4 dpi in the mice upon H1N1 exposure on 7 and 14 dpi of SARS-CoV-2 infection and in the mice without SARS-CoV-2 infection were showed. The data were shown by percentages. (e) CD4+ H1N1-HA-tetramer cells in lung of the mice upon H1N1 exposure on 7 and 14 dpi of SARS-CoV-2 infection and in the mice without SARS-CoV-2 infection were showed. *n *= 3 in every point and the data were analysed using GraphPad Prism 8, and the *P-*values were calculated by one-way ANOVA using SPSS PASW statistical software version 18.0. *0.01 < *P* ≤ 0.05, **0.001 < *P* ≤ 0.01, and ***P ≤ 0.001.

In the process of H1N1 infection, the levels of various lymphocytes were lower in the mice upon H1N1 exposure in the SARS-CoV-2 infection convalescent stage, including CD3 cells and TREG cells in the blood, CD3 cells, Th1, Th2 and Th17 cells in the lung. There were less CD3 cells in PBMC and lung on 2 and 4 dpi in the mice upon H1N1 exposure on 7 and 14 dpi of SARS-CoV-2 infection than in the mice infected with H1N1 without SARS-CoV-2 infection (Figure 6(a,b)). TREG cells in PBMC on 4 dpi in the mice upon H1N1 exposure in the SARS-CoV-2 infection convalescent stage was significantly fewer than that in the mice upon H1N1 exposure without SARS-CoV-2 infection (Figure 6(c)). Th17 cells, Th1 cells and Th2 cells in the lung on 4 dpi in the mice upon H1N1 exposure in the SARS-CoV-2 infection convalescent stage were significantly fewer than those in the mice infected by H1N1 without SARS-CoV-2 infection, except that the levels of Th2 cells in the mice infected by H1N1 on 14 dpi of SARS-CoV-2 infection were similar with ones in the mice infected by H1N1 without SARS-CoV-2 infection (Figure 6(d)). In addition, CD4+ H1N1-HA-tetramer cells were stimulated, and the level of 4 dpi in the mice upon H1N1 exposure on 7 and 14 dpi of SARS-CoV-2 infection were obviously lower than those in the mice without SARS-CoV-2 infection (Figure 6(e)).

According to our results, enhanced infection, more serious injuries were found in the H1N1 infection during the convalescent stage of SARS-CoV-2 infection in hACE2 transgenic mice, possibly due to higher virus loads and lower levels of lymphocytes, and the higher levels of inflammatory cytokine and chemokine could aggravate the lung injury. The mouse model with SARS-CoV-2 and H1N1 infection will be valuable for evaluating antiviral therapeutics and vaccines as well as guiding public health work.

## Discussion

In the past, pandemics of influenza have been caused by the IAV strains H1N1 (1918), H2N2 (1957), H3N2 (1968) and H1N1 (2009) [8]. Currently, we are experiencing the challenge of COVID-19 pandemic, and SARS-CoV-2 or its variant may continuously circulate in the human population in the future. In COVID-19 patients, 94.2% of COVID-19 patients could be coinfected with one or more other pathogens, including 9 viruses, 11 bacteria and 4 fungi [23]. Unfortunately, coinfection of SARS-CoV-2 and IAV has been found in patients reported in China [10], the USA [11], Italy [12], Iran [13], Singapore [14] and other countries. It should be realized that, we are facing the cocirculation of SARS-CoV-2 and influenza viruses in the “twindemic” flu season during the COVID-19 pandemic. Though co-infection with H1N1 and SARS-CoV-2 in hamsters proved that longer SARS-CoV-2 shedding and enhanced lung damage compared to that of SARS-CoV-2 mono-infection [20], it is more important to understand the threat from secondary infection between SARS-CoV-2 and IAV, which may occur frequently in the real world. In the hACE2 transgenic mouse model, we demonstrated H1N1 infection enhancing serious lung damage during the convalescent stage of SARS-CoV-2 infection, with more weight loss and virus shedding, higher cytokines level in the mice recovery from SARS-CoV-2 infection, comparing with the mice infected by H1N1 without SARS-CoV-2 pre-infection.

Profiling serum cytokines in COVID-19 patients revealed that kinds of pro-inflammatory factors and anti-inflammatory factors including TNF-α, IFN-γ, IL-2, IL-4, IL-6 and IL-10 were significantly higher, and IL-6 and IL-10 can be used as predictors for fast diagnosis of patients with a higher risk of disease deterioration [24,25]. Cytokines were also involved in the immune responses to IAVs infections, and substantial increases in IL-6 cytokine levels were observed on 2 days after infection and gradually decreased over the course of infection, IFN-*γ* and IL-10 levels increased on 6 or 7 days after infection [26]. In the inflammatory cytokine and chemokine quantifications, co-infection with H1N1 and SARS-CoV-2 in hamsters found that the abundance of IL-1β, IL-6, TNF-α, IFN-*γ*, mip-1α and RANTES were higher in the co-infection, consistently, more serious histopathology changes in pulmonary oedema, alveolar infiltration and blood vessel inflammation were demonstrated [20]. In our study on H1N1 secondary infection during the convalescent stage of SARS-CoV-2 infection, we found that the abundance of many pro-inflammatory factors such as IL-1β, IL-6,TNF-α and GM-CSF, chemokines such as CXCL-1, CXCL-2, CXCL-5 and CXCL-13, antivirus cytokines such as IFN-βand IFN-*γ*, and anti-inflammatory factors such as IL-4 and IL-10 were higher on 7 dpi after H1N1 infection. These cytokines are produced at sites of tissue inflammation and released into the circulation by a variety of different cell types to regulate the immune responses. IL-6 is an important mediator during acute inflammation and an important cytokine involved in the inflammatory response, playing a role in the induction of acute-phase proteins synthesis and pro-inflammatory effects on a variety of cells[27]. And after H1N1 exposure during the convalescent stage of SARS-CoV-2 infection, IL-6 cytokine levels were increased after infection and the levels were much higher in the mice on 7 dpi infected by H1N1 during the convalescent stage of SARS-CoV-2 infection than ones in the mice infected only by H1N1, and IL-6 cytokine levels were increased earlier in the mice infected by H1N1 on 7 dpi of SARS-CoV-2 infection. These results were consistent with the lung injuries and inflammation responses. On the other hand, IL-10 levels were increased by 7 dpi after infection by H1N1 in the three groups, and the levels were much higher in the mice infected by H1N1 on 7 dpi of SARS-CoV-2 infection than ones in the other two groups. IL-10 is the central anti-inflammatory cytokine, and they can both impede pathogen clearance and ameliorate immunopathology by inhibiting the activity of Th1 cells, NK cells, and macrophages, inhibiting proliferation and production of IL-2, IFN-*γ*, IL-4, IL-5 and TNF-α [28,29]. We assumed that, in the mice which were infected by H1N1 during the convalescent stage of SARS-CoV-2 infection, kinds of pro-inflammatory factors such as IL-6 were increased after H1N1 infection and maybe leaded the serious lung injuries and inflammation, and then, anti-inflammatory factors such as IL-10 were needed and increased to impede pathogen clearance and ameliorate immunopathology. However, the roles and mechanisms in details about the promotions of SARS-CoV-2 infection in the levels of various cytokines in the H1N1 infection needed further experimental verification.

In most patients infected by SARS-CoV-2, the absolute value of lymphocytes is reduced [30], which suggests that SARS-CoV-2 might mainly act on lymphocytes, especially T lymphocytes. In our results, the proportions of CD3 and CD4 cells in PBMC of normal mice were significantly higher than those in the mice upon H1N1 exposure on 7 and 14 dpi of SARS-CoV-2 infection, and only a small minority of lymphocytes could be activated in the SARS-CoV-2 infection but only hold high level for a short time. During H1N1 infection after recovery from SARS-CoV-2, the levels of multiple kinds of lymphocytes were lower, including CD3 cells and TREG cells in PBMC, CD3 cells, Th1 cells, Th2 cells and Th17 cells in the lung. Perhaps the suppression of lymphocytes by SARS-CoV-2 infection caused lower levels of these lymphocytes during H1N1 infection and played roles in aggravating pathological damage from H1N1 infection.

Influenza A may have symptoms similar to those in COVID-19 and lead to complications in the identification and treatment of respiratory syndrome, and the secondary infection between SARS-CoV-2 and IAV could aggravate pathological injury. Countermeasures to prevent and treat secondary infections are a global health priority, and our results will be valuable for evaluating antiviral therapeutics and vaccines as well as guiding public health work.

## Supplementary Material

supplementary_editable_file.docx
