# Supplementary material for: H1N1 exposure during the convalescent stage of SARS-CoV-2 infection results in enhanced lung pathologic damage in hACE2 transgenic mice
Source: Emerg Microbes Infect. 2021 Jun 13;10(1):1156–68. doi: 10.1080/22221751.2021.1938241 (PMC8205102; doi:10.1080/22221751.2021.1938241)
Supplement: supplementary_editable_file.docx [file TEMI_A_1938241_SM3313.docx]

**Supporting information**

**H1N1 exposure during the convalescent stage of SARS-CoV-2 infection results in enhanced lung pathologic damage in hACE2**

**transgenic mice**

Heng Li*^a^, Xin Zhao*^a^, Yurong Zhao* ^a^, Jing Li* ^a^, Huiwen Zheng* ^a^, Mengyi Xue* ^a^,

Lei Guo ^a^, Jian Zhou ^a^, Jinling Yang ^a^, Yuanyuan Zuo ^a^, Yanli Chen ^a^, Zening Yang ^a^,

Qiqi Fan ^a^, Li Qin ^a^, Haijing Shi ^a^, Longding Liu ^a^ ^#^


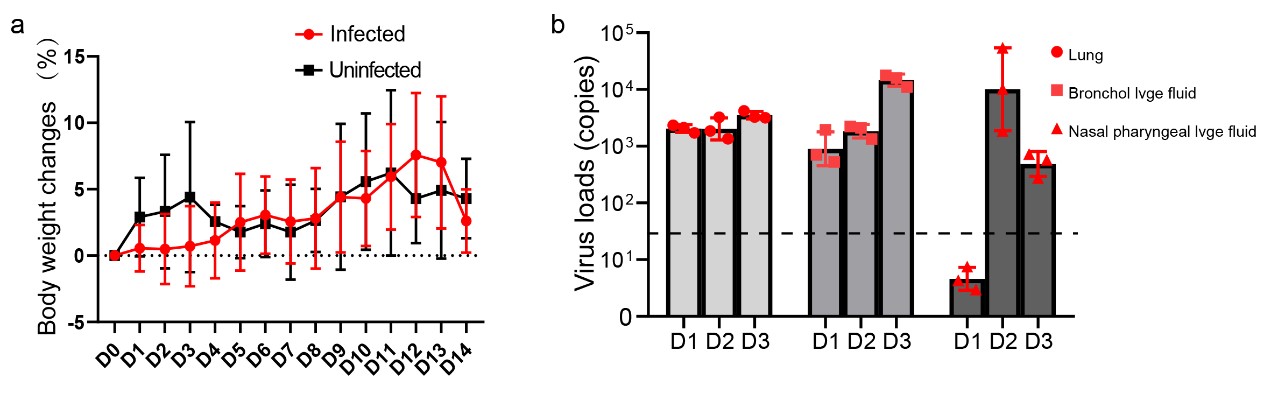


sFIG 1 SARS-CoV-2 infection was validated in hACE2 transgenic mice

(a) Weight changes after challenging with SARS-CoV-2 and the data were showed by change percentages. (b) The virus loads were detected in the VERO cells, and the lung tissue homogenates, BALF and nasal pharyngeal lavage fluid were captured from the mice on 3 dpi infected by SARS-CoV-2 and then incubated in VERO cells for 3 days to detect the virus load.


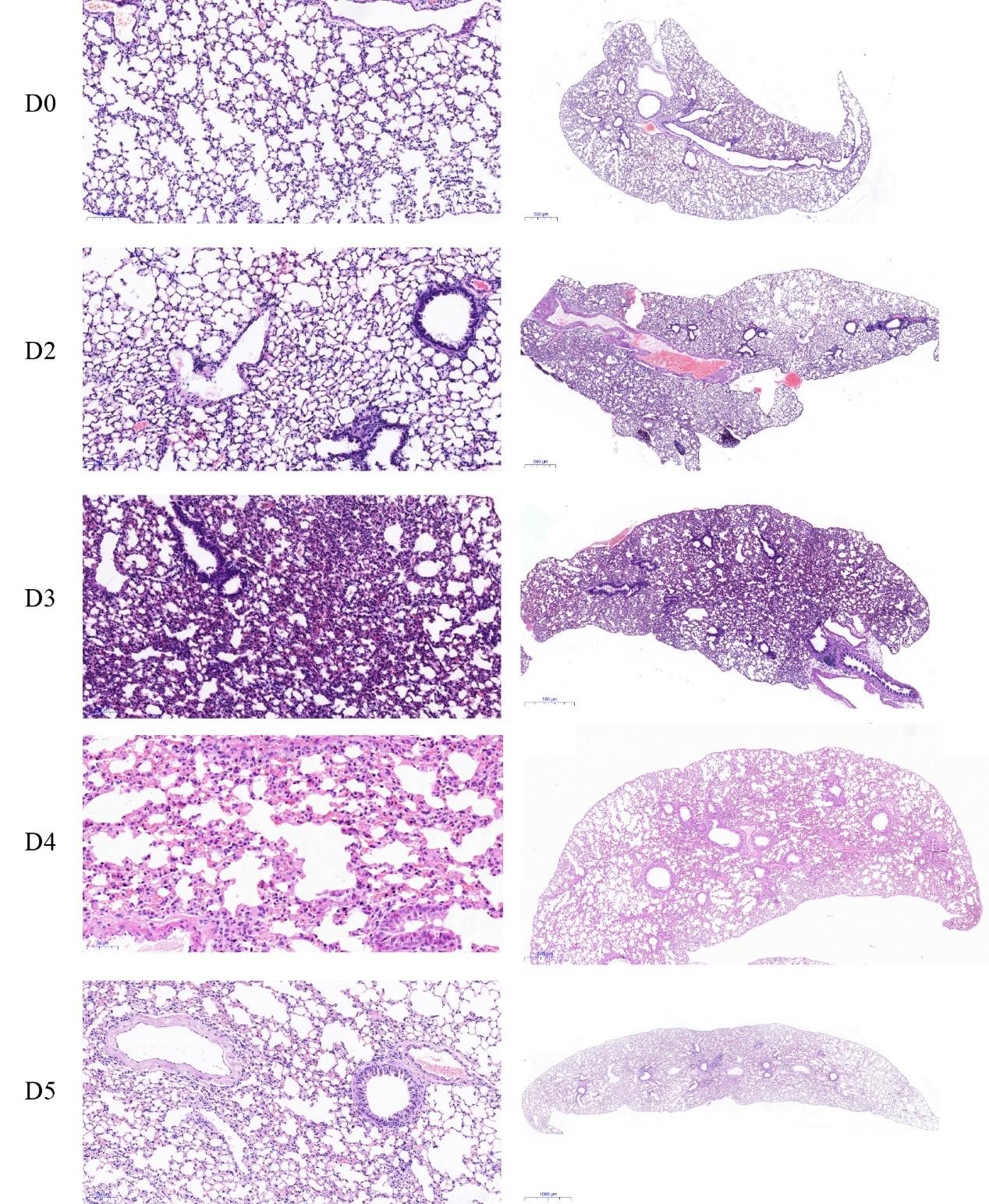


sFIG 2 HE staining examination of the lung in the mice challenged with SARS-CoV-2

On 2 dpi, there were a few infiltrating inflammatory cells and little local pulmonary hemorrhage in the lung. On 3 dpi, some alveolar cells shed, some inflammatory cells infiltrated, and some lung intervals thickened. On 4 dpi, there were some lymphocytes and neutrophil granulocytes in the lung, and the alveoli were slightly dilated. On 5 dpi, there were a few inflammatory cells in the lung, and the lung was similar to the normal lung.


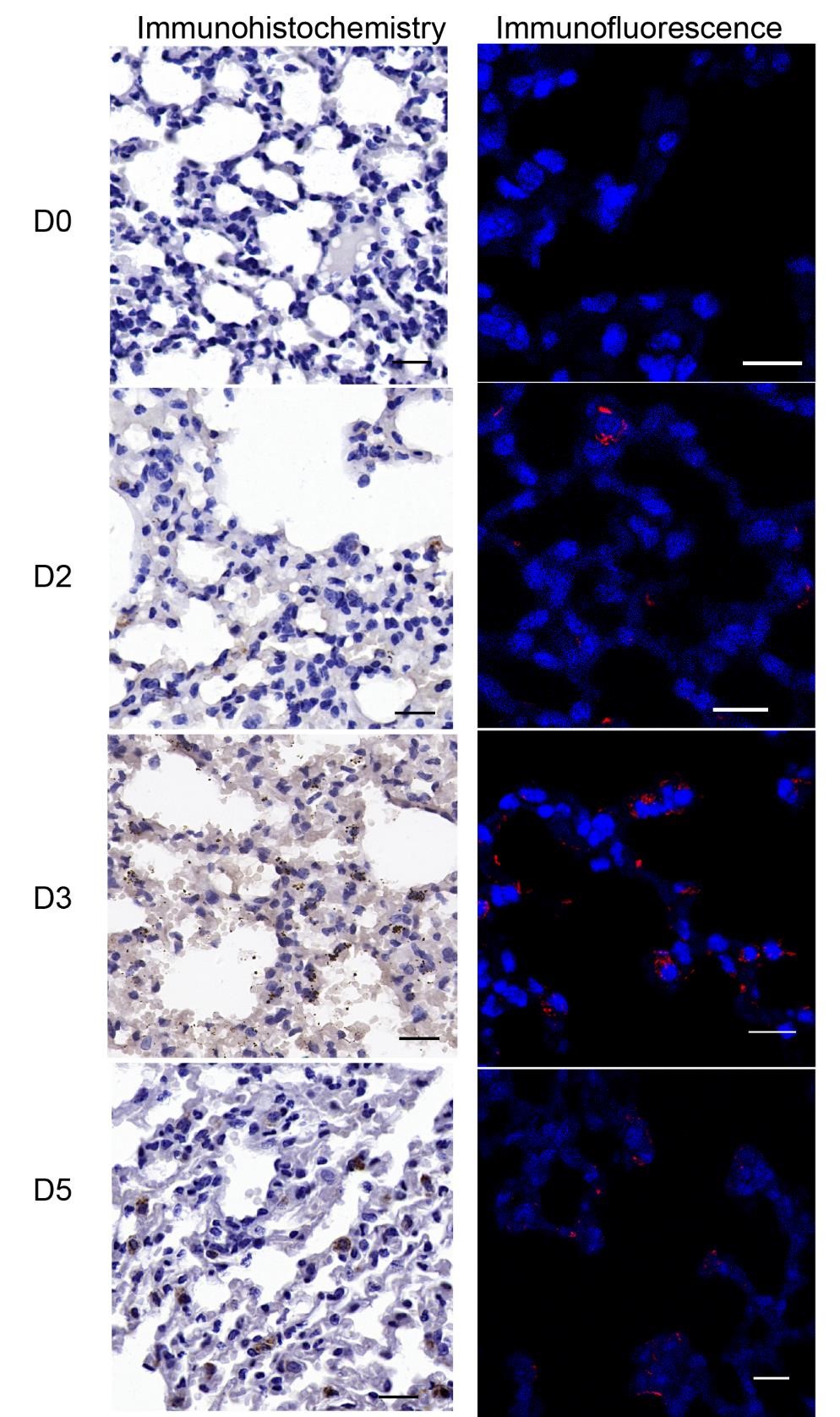


sFIG 3 IHC and IF by anti-SARS-CoV-2 nucleocapsid antibody of the lungs in mice

IHC and IF by anti-SARS-CoV-2 nucleocapsid antibody of the mouse lung on 2, 3 and 5 dpi after infection with SARS-CoV-2. The results showed that the virus level peaked on 3 dpi, and the virus level began to appear on 2 dpi, the virus level on 5 dpi was fewer than those on 3 dpi.


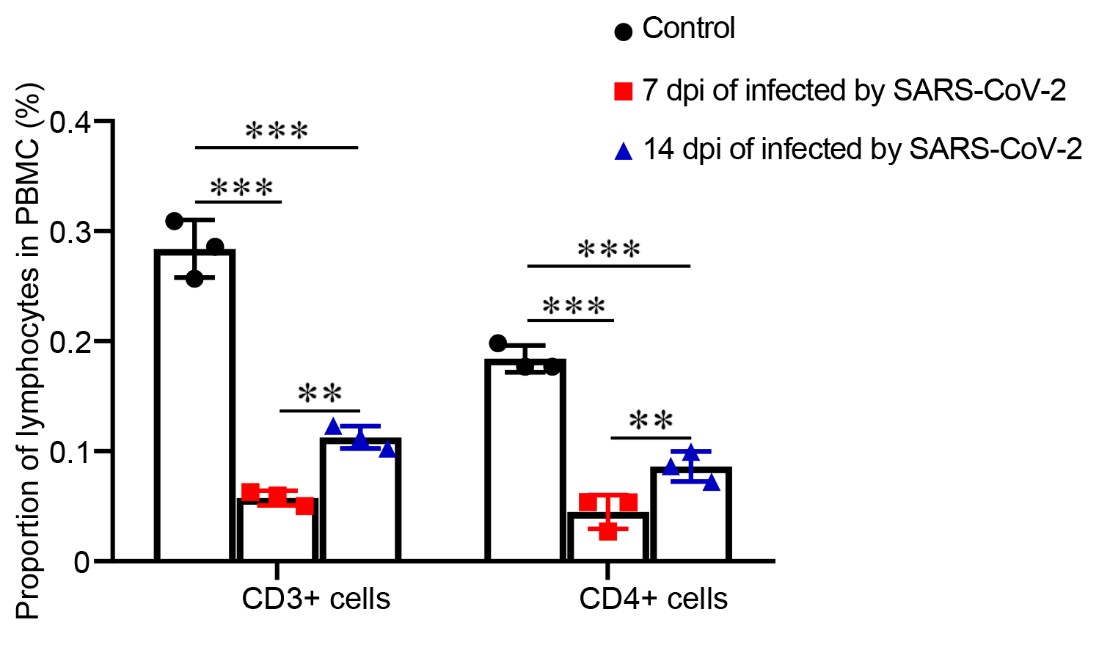


S FIG 4 Comparison of lymphocyte levels during SARS-CoV-2 infection

The proportions of CD3 cells and CD4 cells in PBMC of normal mice were significantly higher than those of the mice infected by SARS-CoV-2 for 7 days and 14 days. *n*=3 in every point and the data were analyzed using GraphPad Prism 8, and the p values were calculated by one-way ANOVA using SPSS PASW statistical software version 18.0. * 0.01 < P ≤ 0.05, ** 0.001 < P ≤ 0.01, and *** P ≤ 0.001.


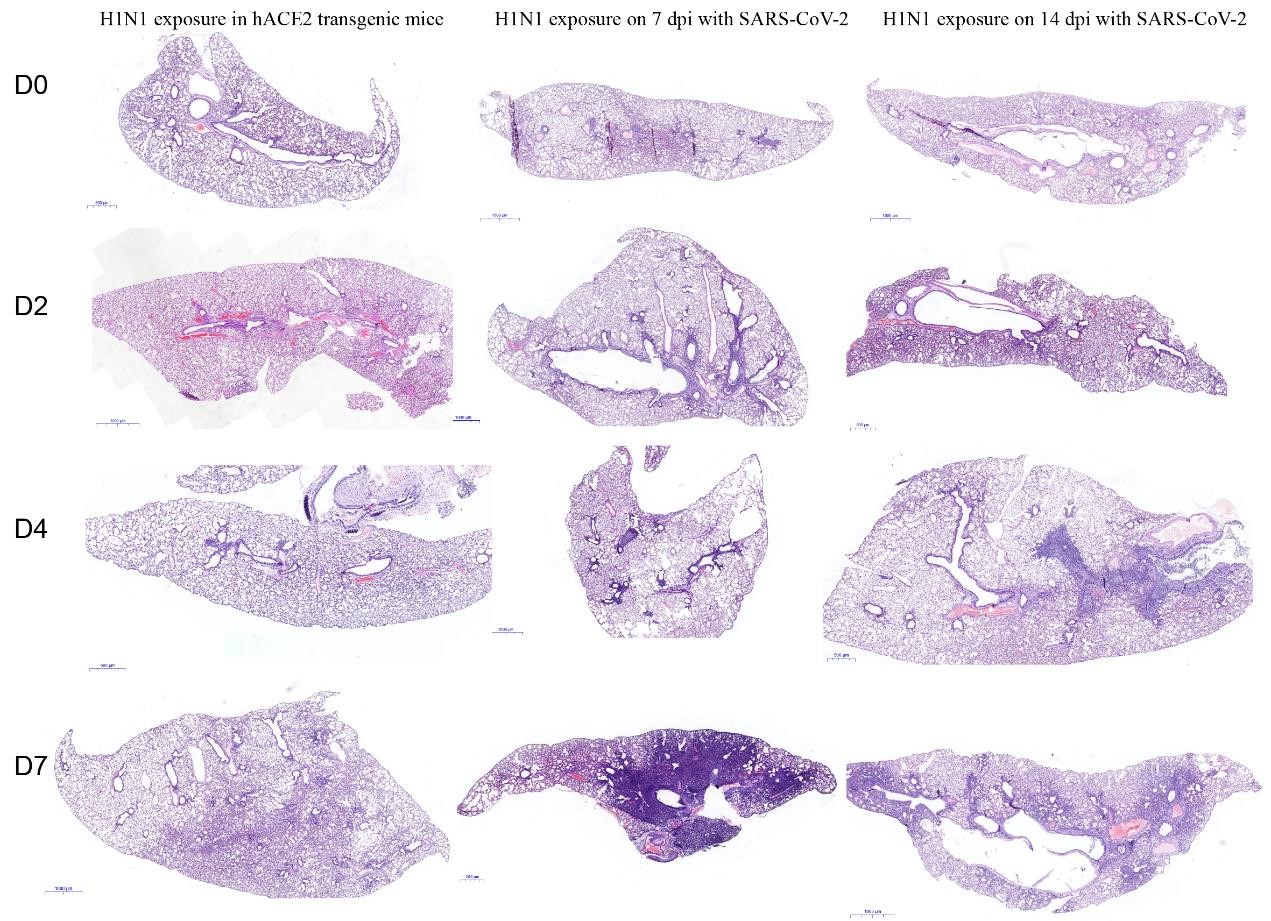


S FIG 5 HE staining examination of the lung in mice infected with H1N1

The lung injury by subsequent H1N1 infection were detected by HE staining, and lung injury by subsequent H1N1 infection was enhanced in the SARS-CoV-2

infection convalescent stage.

# sTable 1. Histology score standards of lung damage

| **Categories** | **Score criteria** | **Score**  **(Total score=12 per animal)**^a^ |
| --- | --- | --- |
| Pulmonary edema | Assessing the location and affecting area in the lung | 1. none 2. alveolar wall edema in one lung lobe^b^ 3. alveolar wall edema in more than one lung lobes 4. diffuse alveolar space edema in one lung lobes 4 diffuse alveolar space edema in more than one lung lobes |
| Alveolar infiltration | Assessing the severity of alveolar septal infiltration and alveolar space infiltration | 1. none 2. peribronchiolar and/or perivascular infiltration 2 peribronchiolar and/or perivascular infiltration and localized alveolar wall infiltration 3. Alveolar space infiltration causing large area tissue consolidation in one lobe 4. diffuse alveolar space infiltration involving more |
|  |  | than one lung lobes |
| Bronchia  infiltration | Blockage area of exudate and exfoliated cells | 1. none 2. small amount of inflammatory exudate around bronchia 3. large local tracheal exudation and slight lumen blockage 4. half of the lumen blockage in one lobe 5. diffuse lumen blockage involving more than one lung lobes |

^a^ Total score = accumulation of scores from individual category assessment ^b^ At least three lung lobes should be examined for each animal

# sTable 2. The primers of 21 kinds of inflammatory cytokine and chemokine and Actin-β

sequence（5’→3’）

Primer name

| mActb-F | GTGACGTTGACATCCGTAAAGA |
| --- | --- |
| mActb-R | GCCGGACTCATCGTACTCC |
| mMCP1-F | TAAAAACCTGGATCGGAACCAAA |
| mMCP1-R | GCATTAGCTTCAGATTTACGGGT |
| mMIP1a-F | TGTACCATGACACTCTGCAAC |
| mMIPla-R | CAACGATGAATTGGCGTGGAA |
| mRANTES-F | TTTGCCTACCTCTCCCTCG |
| mRANTES-R | CGACTGCAAGATTGGAGCACT |
| mCXCL1-F | ACTGCACCCAAACCGAAGTC |
| mCXCL1-R | TGGGGACACCTTTTAGCATCTT |
| mCXCL2-F | CCAACCACCAGGCTACAGG |
| mCXCL2-R | GCGTCACACTCAAGCTCTG |
| mCXCL5-F | GTTCCATCTCGCCATTCATGC |
| mCXCL5-R | GCGGCTATGACTGAGGAAGG |
| mCXCL13-F | GGCCACGGTATTCTGGAAGC |
| mCXCL13-R | ACGACAACAGTTGAAATCACTC |
| mGM-CSF-F | GGCCTTGGAAGCATGTAGAGG |
| mGM-CSF-R | GGAGAACTCGTTAGAGACGACTT |
| mICAM-1-F | TCCGCTACCATCACCGTGTAT |
| mICAM-1-R | TAGCCAGCACCGTGAATGTG |
| mIFN-b-F | AGCTCCAAGAAAGGACGAACA |
| mIFN-b-R | GCCCTGTAGGTGAGGTTGAT |
| mIFN-r-F | GCCACGGCACAGTCATTGA |
| mIFN-r-R | TGCTGATGGCCTGATTGTCTT |
| mIL-2-F | TCTGCGGCATGTTCTGGATTT |
| mIL-2-R | ATGTGTTGTCAGAGCCCTTTAG |
| mIL-4-F | CCCCAGCTAGTTGTCATCCTG |
| mIL-4-R | CAAGTGATTTTTGTCGCATCCG |

| mIL-6-F | CTGCAAGAGACTTCCATCCAG |
| --- | --- |
| mIL-6-R | AGTGGTATAGACAGGTCTGTTGG |
| mIL-10-F | CTTACTGACTGGCATGAGGATCA |
| mIL-l0-R | GCAGCTCTAGGACCATGTGG |
| mIL-12P40-F | GTCCTCAGAAGCTAACCATCTCC |
| mIL-12P40-R | CCAGAGCCTATGACTCCATGTC |
| mlL-17-F | TCAGCGTGTCCAAACACTGAG |
| mIL-17-R | CGCCAAGGGAGTTAAAGACTT |
| mIL-22-F | ATGAGTTTTTCCCTTATGGGGAC |
| mIL-22-R | GCTGGAAGTTGGACACCTCAA |
| mIL-33-F | ATTTCCCCGGCAAAGTTCAG |
| mIL-33-R | AACGGAGTCTCATGCAGTAGA |
| mMMP9-F | GCAGAGGCATACTTGTACCG |
| mMMP9-R | TGATGTTATGATGGTCCCACTTG |
| mTNFa-F | CAGGCGGTGCCTATGTCTC |
| mTNFa-R | CGATCACCCCGAAGTTCAGTAG |

48
